# Anchored linear oligonucleotides: the effective tool for the real-time measurement of uracil DNA glycosylase activity

**DOI:** 10.1098/rsob.210136

**Published:** 2021-10-20

**Authors:** Anna Ligasová, Ivan Rosenberg, Markéta Bocková, Jiří Homola, Karel Koberna

**Affiliations:** ^1^ Institute of Molecular and Translational Medicine, Faculty of Medicine and Dentistry and Czech Advanced Technology and Research Institute, Palacký University Olomouc, 779 00 Olomouc, Czech Republic; ^2^ Institute of Organic Chemistry and Biochemistry, Czech Academy of Sciences, 160 00 Prague, Czech Republic; ^3^ Institute of Photonics and Electronics, Czech Academy of Sciences, 182 51 Prague, Czech Republic

**Keywords:** uracil DNA glycosylase, base excision repair, immobilized oligonucleotides, Förster resonance energy transfer, surface plasmon resonance

## Abstract

Base excision repair is one of the important DNA repair mechanisms in cells. The fundamental role in this complex process is played by DNA glycosylases. Here, we present a novel approach for the real-time measurement of uracil DNA glycosylase activity, which employs selected oligonucleotides immobilized on the surface of magnetic nanoparticles and Förster resonance energy transfer. We also show that the approach can be performed by surface plasmon resonance sensor technology. We demonstrate that the immobilization of oligonucleotides provides much more reliable data than the free oligonucleotides including molecular beacons. Moreover, our results show that the method provides the possibility to address the relationship between the efficiency of uracil DNA glycosylase activity and the arrangement of the used oligonucleotide probes. For instance, the introduction of the nick into oligonucleotide containing the target base (uracil) resulted in the substantial decrease of uracil DNA glycosylase activity of both the bacterial glycosylase and glycosylases naturally present in nuclear lysates.

## Graphical abstract

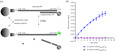

## 1. Introduction

DNA receives endogenous and exogenous insults every minute (approx. 10^4^–10^5^ per cell per day) and the lesions are extremely deleterious to cells [1,2]. These insults involve the action of chemical compounds and various forms of radiation. In addition, the replication errors contribute to the DNA damage during cell proliferation.

Dysregulation of DNA damage response and repair are closely associated with human diseases such as cancers, cardiovascular disease, neurodegenerative disorders and ageing [1–6]. Therefore, effective sensing and repair systems were developed during evolution to eliminate the DNA lesions and to maintain genome integrity [2].

On the other hand, the loss of repair fidelity can be exploited for the treatment of cancer. In this respect, targeting the DNA repair has been an attractive strategy to overwhelm cancer cells with DNA damage, improve the efficacy of radiotherapy and/or chemotherapy, or form part of a lethal combination with a cancer-specific mutation/loss of function [7].

Mismatched and modified DNA bases are primarily processed by base excision repair (BER) pathway through excision and replacement of a single damaged DNA base [8,9]. BER of the damaged bases is initiated by specific DNA glycosylases that recognize and remove the damaged base through the hydrolysis of the *N*-glycosidic bond. There are two different types of DNA glycosylases: monofunctional and bifunctional. Monofunctional DNA glycosylases cleave the *N*-glycosidic bond between the damaged or incorrect nucleobases in the DNA backbone via hydrolytic mechanism, creating an apurinic/apyrimidinic (AP) site. The AP site of the DNA backbone is then recognized and cleaved by AP endonuclease 1 (APE1) providing a 1-nucleotide (nt) gap flanked by 3′-hydroxyl and 2′-deoxyribose-5′-phosphate ends [10,11]. In this respect, an important role for APE1 in telomeric physiology [12] and even in the modulation of gene transcriptional activity or in RNA processing was shown as well [13]. The bifunctional DNA glycosylases can in addition to the damaged base removal cut the phosphodiester bond of DNA, creating a single-strand break. The resulting gap is filled by DNA polymerase [14–16].

One of the frequent DNA damages is the presence of uracil in DNA. It can be a result of deamination of cytosine leading to the creation of a G : U pair or the incorrect incorporation of uracil instead of thymine during DNA replication leading to the creation of an A : U pair. It is not clear how significant the impact of A : U pairs is on DNA structure, however, G : U pairs can lead to the C → T mutations [17]. In mammalian cells, mismatched uracils are recognized and processed by several monofunctional DNA glycosylases, such as uracil-*N*-glycosylase (mitochondrial UNG1 and nuclear UNG2), single-strand selective monofunctional uracil-DNA glycosylase 1 (SMUG1), thymine–DNA glycosylase (TDG) and methyl-binding domain 4 protein (MBD4) [18]. UNG2 is most highly expressed in the S phase of the cell cycle and has an important function both in the post-replicative repair of A : U and G : U pairs [18,19]. TDG has the highest expression in G1 phase [17] and its most efficiently processed substrate is a G : U mispair [20]. SMUG1 is not cell cycle regulated [21] and seems to play an important role in the repair of 5-hydroxymethyl uracil (HmU) [18]. It is supposed that all the mentioned glycosylases are also able to recognize 5-fluorouracil (FU) [18] although UNG2 seems to be responsible for this repair at least in some human cell lines [22]. Since the incorporation of uracil instead of thymine is very common, UNG glycosylases present crucial enzymes in maintaining genome integrity.

It emerged that UNG is an attractive target in lung cancer treatment as BER inhibition could prevent the development of tumours resistant to treatment based on folate analogues [23,24]. It is evident that studies focusing on the analysis of repair systems, including studies dealing with the treatment of cancer, require the existence of reliable approaches for measurement of involved enzymes activities.

The measurement of UNG activity can be realized by various strategies [25–36]. They include approaches based on the combination of FAM-labelled hairpin DNA probe, exonuclease treatment and graphene-induced quenching [30], employing G-quadruplex formation [32,33] or using single-molecule detection [34]. In addition, fluorescence-based method employing both glycosylase and AP endonuclease activities was used for the investigation of the overall activity of those enzymes in cell extracts [35].

Most of these methods are relatively time-consuming, performed in several steps, require additional enzyme activities or do not allow real-time monitoring of glycosylase activity. The approach based on molecular beacons using Förster resonance energy transfer (FRET) for the measurement of DNA glycosylase activity is the exception [28] as no additional components are necessary, the procedure is fast and evaluation can be performed by common plate readers. Molecular beacons are composed of a single-stranded oligonucleotide probe containing at both ends short self-complementary sequences with the target base (e.g. uracil) and fluorochrome and quencher at the 5′- and 3′-ends, respectively. After the cleavage of the target site by DNA glycosylase, the stem of the beacon becomes unstable and undergoes denaturation upon increasing fluorescence [28]. On the other hand, the structure of molecular beacons makes this approach less suitable for the analysis of structural aspects such as the impact of the presence of single-stranded DNA stretches or the presence of the break in DNA on the uracil glycosylase activity.

In this study, we present two effective methods for the determination of uracil DNA glycosylase activity. The first one is based on fluorescently labelled oligonucleotides anchored to the surface of streptavidin-coated magnetic beads, the second on non-labelled oligonucleotides anchored to a streptavidin-coated surface plasmon resonance-based sensor. Both systems allow one-step real-time studies. We evaluated the proposed methods with both the bacterial UNG and glycosylases naturally occurring in cell lysates. We also performed the simultaneous analysis of the UNG content in cell lysates. In addition, we compared the results with those obtained using molecular beacons and non-immobilized oligonucleotide probes.

## 2. Material and methods

### 2.1. Magnetic sensors and oligonucleotide probes

#### 2.1.1. Description

For the construction of the magnetic sensors (m-sensors), double-stranded oligonucleotide (ON) probes were coupled to the streptavidin-coated magnetic particles (NanoLink Streptavidin Magnetic Beads, 10 mg ml^−1^, TriLink Biotechnologies) via biotin. As stated by the manufacturer, the diameter of the magnetic particles is approximately 1 µm, and the binding capacity is greater than 2.5 nmol mg^−1^ of the biotinylated ON. Sequences of the oligonucleotides used for the construction of the probes are listed in table 1.

**Table 1.** List of the oligonucleotides used.

name	5′ modif.	5′ → 3′ direction	3′ modif.
anchoring oligonucleotides			
A_30FAM_	biotin	CGC CTA CAG CAG CGC CAA ATT CTT AAG TGC	6-FAM
B_30FAM_	6-FAM	CGT GAA TTC TTA AAC CGC GAC GAC ATC CGC	biotin
B_30_	—	CGT GAA TTC TTA AAC CGC GAC GAC ATC CGC	biotin
B_14FAM_	6-FAM	CGT GAA TTC TTA AA	biotin
B_14_	—	CGT GAA TTC TTA AA	biotin
B_30U_	—	CGT GAA T**U**C TTA AAC CGC GAC GAC ATC CGC	biotin
complementary oligonucleotides			
C_XQ_^a^	—	TTT AAG AA**X** TCA CG	BHQ1^b^
C_4UQ_	—	T**UU** AAG AA**U** **U**CA CG	BHQ1^b^
D_UQ_	BHQ1^b^	GCA CT**U** AAG AAT TT	—
C_U_	—	TTT AAG AA**U** TCA CG	—
other oligonucleotides			
E_Cy5_	Cy5	GTT GCC TTA GGT TTT TCG TCG ATT TTT CCT TAG GTT TTT CGT CGA T	biotin
F_break_	—	GCG GAT GTC GTC GCG G	—

^a^**X** = thymine (T) or uracil (**U**).

^b^BHQ1 = quencher suitable for fluorochromes emitting in the wavelength range 480–580 nm, absorption maximum is 534 nm.

The used ONs were either purchased from Generi Biotech or synthesized in Dr Rosenberg IOCB laboratory (for details see electronic supplementary material, methods 1.1).

To compensate for imperfections during preparation, m-sensors concurrently containing ON with Cy5 fluorochrome (E_Cy5_, Generi Biotech; table 1) were employed as an internal standard. For the determination of the impact of the break on the speed of uracil cleavage, the ON probe F_break_ was used (Generi Biotech; table 1). The ONs shown in table 2 (Generi Biotech) were used for competitive analysis.

**Table 2.** List of the oligonucleotides used for the competitive analysis.

name	5′ → 3′ direction
oligo_9_U	GCA GT**U** AGA
oligo_12_U	GCA GT**U** AGA TCA
oligo_15_U	GCA GT**U** AGA TCA TCG
oligo_18_U	GCA GT**U** AGA TCA TCG CAG
oligo_9_T	GCA GTT AGA
oligo_12_T	GCA GTT AGA TCA
oligo_15_T	GCA GTT AGA TCA TCG
oligo_18_T	GCA GTT AGA TCA TCG CAG

The advantage of the magnetic beads compared to the non-magnetic ones is their simple separation using magnetic forces enabling quick incubation and washing. Even particles with a size of around several tens of nanometres can be separated very quickly and efficiently. Although the magnetic separator is required, it is relatively cheap and it usually enables simultaneous separation of many samples.

#### 2.1.2. Modification of magnetic particles with oligonucleotide probes

The streptavidin-coated magnetic particles were first incubated with biotinylated anchoring ON and then hybridized with the complementary ON. In particular, one volume of the suspension of magnetic particles (10 mg ml^−1^) was diluted in 10 volumes of TBT buffer (25 mM Tris–HCl, pH 7.5, 150 mM NaCl, 0.1% BSA, 0.05% Tween and 0.02% sodium azide) and particles were separated with UniTrap magnetic separator (it allowed particle separation in the narrow visible band even when using concentration of m-sensors as low as 1 µg ml^−1^; http://transfer.vtpup.cz/unitrap/). After separation, the particles were washed twice with TBT buffer, resuspended in 10 volumes of TBT buffer containing 2.5 µM biotinylated anchoring ON and incubated for 1 h on vortex (300 r.p.m.). In this respect, the half concentration of the biotinylated anchoring ON provided lower signal and the double concentration did not lead to a signal increase (electronic supplementary material, figure S1). In m-sensors with internal standard, E_Cy5_ ON was added to the mixture with the biotinylated anchoring ON at a concentration of 0.25 µM to quantify the added amount of magnetic particles per sample. This concentration provided sufficient signal and did not substantially prevent the binding of biotinylated anchoring ON. M-sensors with biotinylated anchoring ON were washed three times with 10 volumes of TBT buffer. Then, m-sensors were incubated with 10 volumes of TBT buffer with 5 µM complementary ONs on vortex (300 r.p.m.) for 1 h. Finally, m-sensors were either immediately used or stored at 4°C for up to one month. The approximate amount of the probes bound to one magnetic particle was 4.8 × 10^6^ of the probe on average (see electronic supplementary material, methods 1.2).

### 2.2. Measurement of uracil DNA glycosylase, DNase I and exonuclease III activity by m-sensors

One volume of the prepared m-sensors was washed with 100 volumes of TBT buffer. After magnetic separation, m-sensors were washed three times with 100 volumes of (i) the NaCa buffer (2 mM CaCl_2_, 50 mM NaCl, 10 mM Tris–HCl, pH 7.4 and 1 mM EDTA) for the analysis of uracil DNA glycosylase activity, (ii) the DNase I buffer (100 mM Tris–HCl, pH 7.4, 50 mM NaCl, 2.5 mM MgCl_2_ and 1 mM CaCl_2_) for the analysis of DNase I activity or (iii) the exoIII buffer (66 mM Tris, pH 8, 50 mM NaCl and 2 mM MgCl_2_) for the analysis of exonuclease III activity. The optimal composition of the buffer for the analysis of uracil DNA glycosylase was tested at first (for details see electronic supplementary material, methods 1.3). Subsequently, 50 µl of the washed m-sensors (approx. 0.5 µg of magnetic particles, for more details about the used amount of m-sensors, see electronic supplementary material, results 2.1, figures S2 and S3) were used per well of the black 384-well plate. Then, 50 µl of the enzyme solution in the same buffer were added. The tested enzymes were bacterial uracil DNA glycosylase (UNG, source *Escherichia coli* K12 cells, ThermoFisher Scientific, EN0361), DNase I (ThermoFisher Scientific, EN0525) and exonuclease III (ThermoFisher Scientific, EN0191). If the cell lysates were tested, the final concentration of the prepared nuclear or cytoplasmic lysates (overall proteins) added per well was 2 or 4 µg ml^−1^. The fluorescence signal was measured in 2 min intervals if not stated otherwise. Before measurement, the well plate was tempered in the plate reader to 25°C for 4 min.

In the case of bacterial UNG, the initial rate of the signal growth (the speed of the signal growth) was determined as a value of a first derivation of the regression function of the dependence of the signal's quotient measured for 6-FAM and Cy5 on the time at the beginning of the reaction if not stated otherwise. In the case of measurement of uracil glycosylase activity in cell lysates, DNase I and exonuclease III activity, the rate of the signal growth was calculated as a value of a first derivation of the regression function of the dependence of the signal measured for 6-FAM at the beginning of the reaction.

### 2.3. SPR sensor and assay

The SPR sensor was primarily employed to investigate the effect of the double-stranded probe design on the activity of UNG glycosylase. In particular, a laboratory SPR platform based on the wavelength spectroscopy of surface plasmons (Plasmon IV) [37] was used combined with dispersionless microfluidics [38], both developed at the Institute of Photonics and Electronics, Czech Republic. The SPR assay optimization is described in electronic supplementary material, methods 1.4. All the experiments were performed at a temperature of 25°C and under a flow rate of 20 µl min^−1^. The immobilization of biotinylated anchoring ON probes onto the surface of the SPR sensor chips was achieved via streptavidin covalently attached to a ω-carboxyalkylthiol self-assembled monolayer (SAM). The preparation of mixed SAMs of HS–C_11_–(EG)_4_–OH and HS–C_11_–(EG)_6_–OCH_2_–COOH alkylthiols and *in situ* immobilization of the streptavidin are described in detail in [39]. In order to provide reproducible binding conditions, the same amount of streptavidin (1.7 × 10^12^ streptavidin cm^−2^, corresponding to an SPR sensor response of 10 nm) was immobilized on the surface of an SPR chip prior to each experiment. As sensor surface functionalization and receptor attachment have direct impact on the final sensor performance [40], the SAM of alkylthiolates was employed as it provides much more robust functional coating and protection against non-specific adsorption from complex media that, for example, just direct chemisorption of receptors via thiol groups. Another reason for using attachment of biotinylated oligonucleotides to streptavidin-coated sensor surface was to employ as similar an approach to the one used with m-sensors as possible.

After the immobilization of streptavidin, the attachment of biotinylated anchoring ON probes, subsequent hybridization with the complementary ONs and application of UNG was performed as follows: TrisNa buffer (10 mM Tris, 150 mM NaCl, pH 7.4) was flowed along the sensor surface to establish the baseline. Subsequently, the sensor surface was exposed to 100 nM solution of biotinylated anchoring ON probe until the desired surface probe coverage was achieved (approx. 5 min) after which TrisNa buffer was injected for 10 min. Then, a solution of 100 nM complementary ON was flowed along the sensor surface until the desired number of duplexes was formed (approx. 10 min), followed by TrisNa buffer again. Finally, NaCa buffer was flowed until a stable baseline was achieved and UNG solution (1 : 5000 diluted, corresponds to 200 mU ml^−1^) was injected for 15 min, followed by NaCa buffer again. The sequences of the ONs used are listed in table 1.

In the experiments, in which single-stranded probe arrangement was investigated, the step containing complementary ON was excluded, whereas in experiments with DNA-chain-interruption-containing probes, an extra step with F_break_ complementary ON was added prior to the injection of NaCa buffer-UNG-NaCa buffer sequence.

### 2.4. Cell cultivation

The HeLa cells (cervix, adenocarcinoma, a gift from Dr David Staněk, Institute of Molecular Genetics CAS, Prague) were cultivated in Dulbecco's modified Eagle's medium (DMEM) supplemented with 10% fetal bovine serum, 3.7 g l^−1^ of sodium bicarbonate and 50 µg ml^−1^ of gentamicin. The cells were cultivated at 37°C in a humidified atmosphere containing 5% CO_2_. The cell line was regularly tested for mycoplasma contamination by PCR and enzymatic detection [41].

### 2.5. Preparation of cell lysates and the protein content determination

Nuclear and cytoplasmic fractions of the cell lysates were used in experiments and were prepared as follows: the HeLa cells were plated onto the Petri dish (100 mm in diameter) and cultivated until 70–80% confluence. The cells were washed with 1 × PBS buffer and then quickly rinsed with the ice-cold buffer composed of 10 mM Tris–HCl, pH 7.4 and 10 mM KCl (buffer A). The ice-cold buffer B composed of 10 mM Tris–HCl, pH 7.4, 10 mM KCl, 0.5 mM EDTA and protease inhibitors (Sigma Aldrich) was subsequently added (2 ml) and the cells were incubated on ice for 20 min. The cells were scratched using the cell scraper, transferred to the ice-cold tempered Dounce homogenizer and homogenized (30 times). The homogenate was transferred into the 2 ml tube and centrifuged 10 min at 12 000*g*, 4°C. The liquid cytoplasmic fraction was transferred to the new, ice-cold tube and used for measurements or divided into aliquots and stored at −80°C. Then, 50 µl of buffer B was added to the pellet, the pellet was resuspended; 200 µl of buffer C (0.5 M KCl, 10 mM Tris–HCl, pH 7.4, 0.5 mM EDTA and protease inhibitors) were added, mixed on laboratory vortex and incubated on ice for 40 min. The samples were briefly mixed every 10 min. Further, the samples were centrifuged 20 min at 12 000*g*, 4°C. The supernatant (nuclear fraction) was transferred to the new ice-cold tube and either used or aliquoted and stored at −80°C.

In some experiments, Triton X-100 was used during the preparation of the cell lysates (for details see electronic supplementary material, methods 1.5).

The protein content was determined in both fractions using the Pierce BCA protein assay kit (ThermoFisher Scientific) according to the manufacturer's protocol.

### 2.6. SDS–PAGE, western blot

In order to evaluate the amount of UNG in cell lysates, the SDS–PAGE and western blots were performed according to [42]. Briefly, 1 µg of the total protein if not stated otherwise was resolved by SDS–PAGE at a constant voltage of 100 V for the first 10 min and 120 V for 1 h and 50 min. The proteins were then transferred to a nitrocellulose membrane (0.2 µm pore size, Bio-Rad) using the TE 22 Mighty Small Transfer Tank (Hoefer). The membrane was blocked in 5% BSA in TBS/T (Tris-buffered saline with 0.1% Tween-20) for 1 h and incubated with primary antibody against UNG (OriGene, TA503563) in 5% BSA and TBS/T overnight at 4°C with agitation. Then, the membranes were washed with TBS/T and incubated with peroxidase-labelled secondary antibody. The membranes were washed and incubated briefly with Luminata Forte peroxidase substrate (Merck). The chemiluminiscence was collected by the ChemiDoc MP Documentation system (Bio-Rad). The data were evaluated using ImageJ and Microsoft Excel software [42].

### 2.7. Suppression of UNG expression

The expression of UNG was decreased by siRNA as described in [42]. UNG-specific and control siRNAs consisting of pools of three target-specific or non-specific 19–25 nt siRNAs (Santa Cruz Biotechnology, sc-37803) were used. The HeLa cells were treated with 50 nM siRNA for 7 h, then the culture medium was added to the transfection solution and the cells were further incubated for 24 h. Further, the culture medium with transfection solution was exchanged for the normal culture medium and the cells were incubated for an additional 72 h [42]. After transfection, the cytoplasmic and nuclear lysates were prepared.

### 2.8. Data acquisition and evaluation

In the case of black 384- and 96-well plates, the fluorescence signal was measured using the Infinite 200 Pro Plate Reader (Tecan). Fluorescence of 6-FAM was measured at 488 nm excitation wavelength and 520 nm emission wavelength, while the fluorescence of Cy5 (used as an internal standard) was measured at 630 nm excitation wavelength and 680 nm emission wavelength. The signal was measured every 2 min in 10 cycles if not stated otherwise. Before each measurement, the samples were shaken by the plate reader (10 s, 1.5 mm amplitude). We performed the evaluation of m-sensors analysis using fluorescence microscopy as well (for details, see electronic supplementary material, methods 1.6, results 2.2, videos S1 and S2, and figure S5).

**Figure 1.**
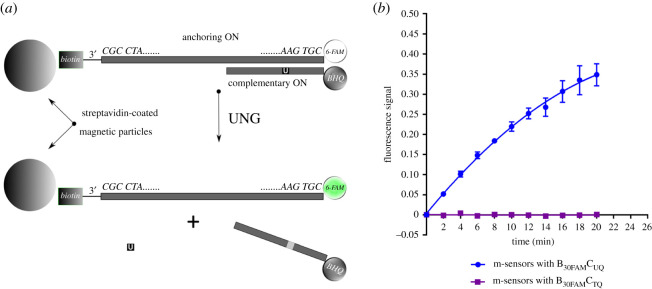
(*a*) The scheme of the method principle is shown. The probes composed of the anchoring and complementary ONs are anchored on the magnetic particles coated with streptavidin (m-sensors). The anchoring ON contains biotin on its 3′ and 6-FAM fluorochrome on 5′ end. The complementary ON contains uracil (U) and the fluorescence quencher BHQ-1 on its 3′ end. During incubation with UNG, uracil is cleaved and the chains are separated. It results in the separation of the ON strands with BHQ-1 and 6-FAM and an increase of the fluorescence. (*b*) An example of the measurement of glycosylase activity using m-sensors with B_30FAM_C_UQ_ a B_30FAM_C_TQ_ probes and bacterial UNG is shown. The fluorescence signal of 6-FAM and Cy5 was acquired every 2 min. The signal for 6-FAM was divided by the Cy5 signal. The data are shown as the mean ± s.d.

**Figure 2.**
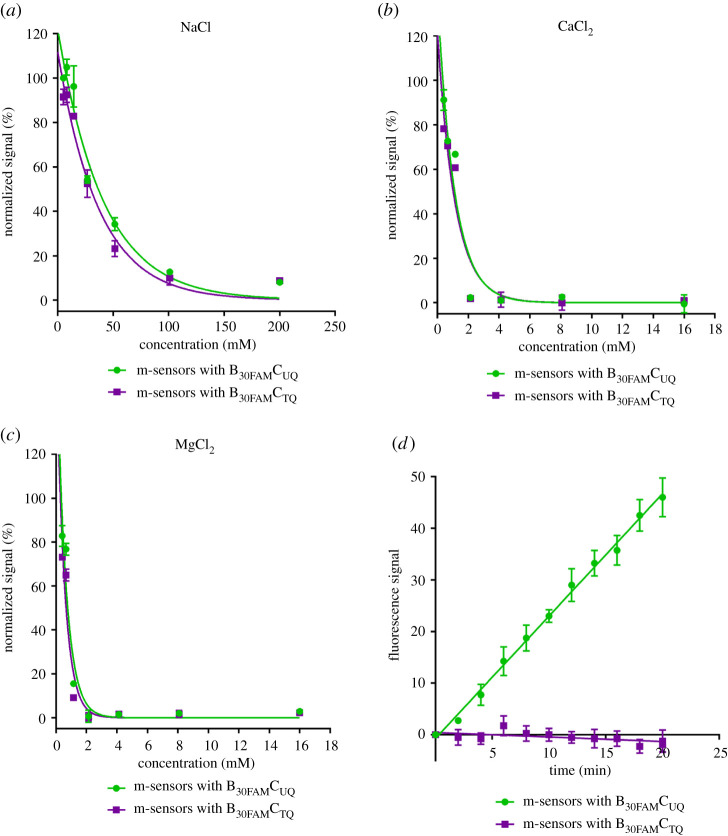
The impact of ions on the ON probe stability. (*a*–*c*) The dependence of the signal of m-sensors with the ON probe with uracil (B_30FAM_C_UQ_) or with thymine (B_30FAM_C_TQ_) on the concentration of NaCl (*a*), CaCl_2_ (*b*) and MgCl_2_ (*c*). M-sensors were incubated in solutions containing various concentrations of the above-mentioned ions for 30 min. Then, the signal of 6-FAM was measured. The signal is normalized to the value measured for 5.09 mM NaCl concentration equal to 100%. The data are shown as the mean ± s.d. (*d*) The determination of glycosylase activity in cell lysates using the NaCa buffer is shown. The m-sensors with B_30FAM_C_UQ_ or B_30FAM_C_TQ_ were used. The fluorescence signal of 6-FAM and Cy5 was acquired every 2 min. The data are shown as the mean ± s.d.

The obtained data were analysed using CellProfiler [43,44], ImageJ and Microsoft Excel software and the final graphs were plotted in GraphPad Prism 6 [41]. The graphs were constructed using the following functions: one-phase decay was used in the case of the analysis of data in figure 2*a–c*, one-phase association was used for the analysis of data in electronic supplementary material, figure S3, second-order polynomial (quadratic) regression was used for the analysis of the data in figures 1*b* and 8*a*–*c* (except m-sensors with T in 8*a*,*b*), and electronic supplementary material, figures S1, S2 (6-FAM/Cy5) and S5 (probe B_30FAM_C_UQ_). Linear regression was used for the analysis of the data in figures 2*d*, 8*a*,*b* (m-sensors with T) and 5*b*, and electronic supplementary material, figures S2 (6-FAM) and S5 (probe B_30FAM_C_TQ_).

**Figure 5.**
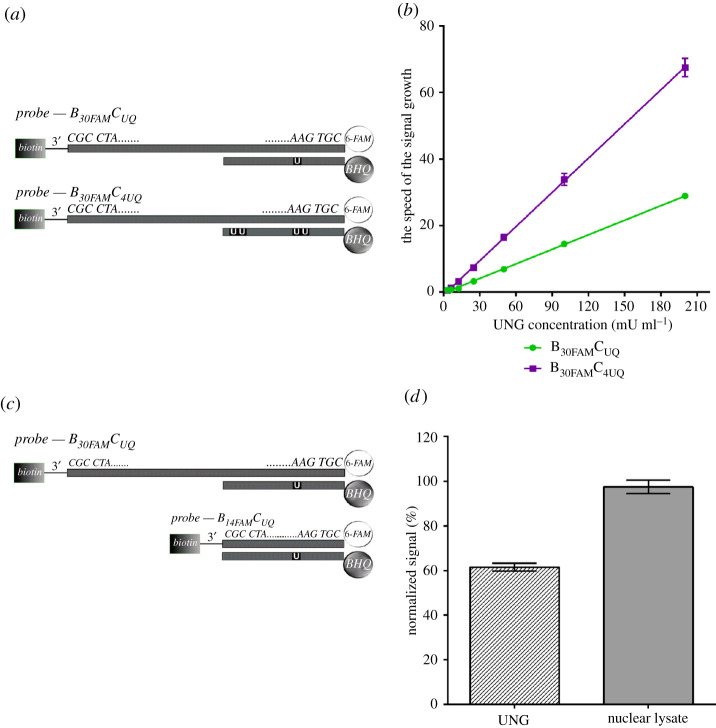
The impact of the number of uracils and the length of the anchoring oligonucleotide. (*a*) Scheme of ON probes used for the analysis of the impact of the number of uracil on glycosylase activity. (*b*) The results of the measurement of the speed of the signal growth with m-sensors containing probes with one or four uracils in solutions with various concentration of bacterial UNG; 0.5 µg of m-sensors was added to the solution of bacterial UNG. The concentration of UNG was alternatively 200, 100, 50, 25, 12.5, 6.25 or 3.125 mU ml^−1^. The data are shown as the mean ± s.d. (*c*) The scheme of ON probes used for the analysis of the impact of the probe length on glycosylase activity. (*d*) The results of the measurement of the speed of the signal growth of m-sensors with the anchoring ON containing 30 or 14 nt. M-sensors were incubated with the bacterial UNG or the nuclear lysate of HeLa cells (2 µg ml^−1^ of the overall protein). The speed of the signal growth was normalized to the speed of the signal growth of m-sensors containing anchoring ON with 30 nt (equal to 100%). The data are shown as the mean ± s.d.

**Figure 8.**
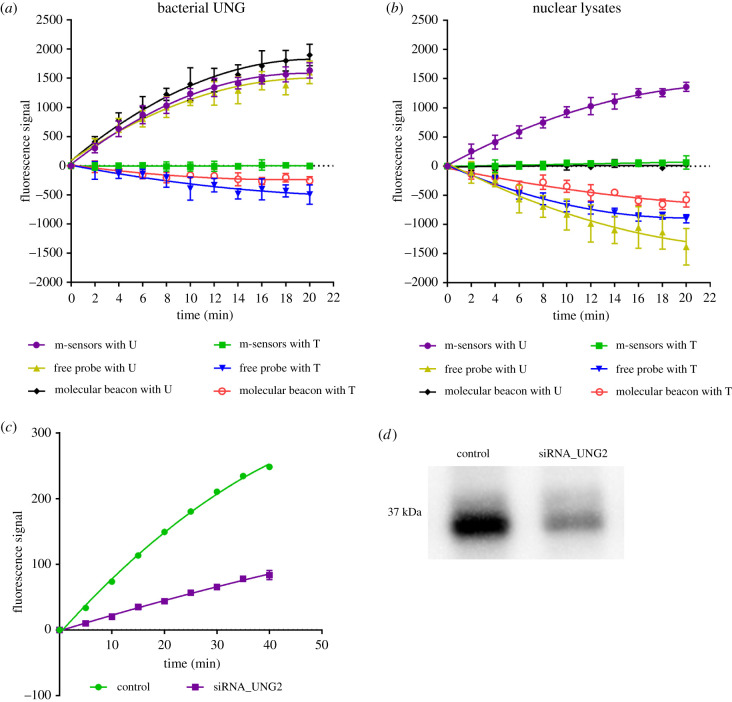
Comparison of m-sensors, free probes and molecular beacons and the effect of UNG level decrease. (*a*) Measurement of glycosylase activity in the solution of bacterial UNG is shown. M-sensors or free probes or molecular beacons were incubated with the bacterial UNG. The fluorescence signal of 6-FAM was acquired every 2 min. The data are shown as the mean ± s.d. (*b*) Measurement of glycosylase activity in nuclear lysates (4 µg ml^−1^ of the overall proteins) is shown. M-sensors or free probes or molecular beacons were incubated with the solution of nuclear lysate of HeLa cells and the fluorescence signal of 6-FAM was measured in 2-min intervals. The data are shown as the mean ± s.d. (*c*) Impact of lowering of UNG level on the signal provided by m-sensors in nuclear lysates of HeLa cells. HeLa cells were incubated with UNG-siRNA or with the control siRNA and then the nuclear lysates were prepared. M-sensors with B_30FAM_C_UQ_ or B_30FAM_C_TQ_ probes were added to the nuclear lysates (2 µg ml^−1^ of the overall protein) and the signal was measured in 2 min intervals. The value of the signal is equal to the difference of the signal from probes with uracil and with thymine. The data are shown as the mean ± s.d. (*d*) western blot analysis of the content of UNG in HeLa cells with control siRNA and UNG-siRNA. One microgram of the total protein was resolved by SDS–PAGE at 100 V for the first 10 min and 120 V for 1 h and 50 min. The proteins were then transferred to a nitrocellulose membrane (75 V, 60 min).

All the performed experiments were done in three independent replicates. The data are presented as the mean values ± standard deviation (s.d.).

Inkscape software was used to draw the schemes and Adobe Photoshop was used to prepare figures.

## 3. Results and discussion

### 3.1. The method principle

Two types of methods were used to investigate the glycosylase activity. Both were based on the ON duplex attached to the streptavidin-coated sensor surface via biotin. In the first case, the ON probes were anchored to the magnetic particles (m-sensors), in the second case, to the chip of an SPR sensor.

#### 3.1.1. M-sensors

The scheme of the basic ON arrangement used with m-sensors is shown in figure 1*a*. The anchoring ON contained biotin and fluorochrome 6-FAM situated on the opposite ends of the chain. The ON complementary to the anchoring ON contained Black Hole Quencher-1 (BHQ-1) and the uracil target base. In the control complementary ONs, uracil was replaced with thymine. When the m-sensors with the target uracil were placed into solutions containing enzymes with glycosylase activity and the target uracil was cleaved, the abasic site emerged causing the destabilization of the ON duplex. Then, the 6-FAM fluorochrome was separated from the BHQ-1 quencher and the fluorescence signal was recorded as a function of time. In the case of m-sensors, the increase of the fluorescence was measured in black 96- or 384-well plates using the plate reader or by fluorescence microscopy (for microscopy measurements, see electronic supplementary material, methods 1.6, results 2.2, videos S1 and S2, and figure S5). The specificity of the method was verified using the control ON probes with thymine. Typical temporal fluorescence signal obtained for bacterial UNG and m-sensors with B_30FAM_C_UQ_ and B_30FAM_C_TQ_ probes using plate reader is shown in figure 1*b*.

The buffer composition for the glycosylase activity measurement was optimized both in terms of the stability of the probes and in terms of its impact on the glycosylase and nuclease activity.

M-sensors with probes containing either uracil (B_30FAM_C_UQ_) or thymine (B_30FAM_C_TQ_) and buffers containing various concentrations of CaCl_2_ or MgCl_2_ or NaCl were used (for details see electronic supplementary material, methods 1.3). The stability of the ON probe duplexes (indicated by low fluorescence) was the highest in solutions containing MgCl_2_ and the lowest in solutions with NaCl (figure 2*a*–*c*; electronic supplementary material, table S1). Probes with uracil exhibited a slightly higher signal in the same solution than the probes with thymine.

The tested concentrations of MgCl_2_ and CaCl_2_ had no significant impact on the measurement of the activity of bacterial UNG. To analyse the impact of the buffer composition on the activation of cellular nucleases, the stability of the probes with thymine was followed in the nuclear lysates of HeLa cells. The obtained data showed that the solutions with MgCl_2_ strongly activated nuclease activity in nuclear lysates and caused the rapid degradation of probes. Only very low nuclease activity was observed if the buffer was supplemented with CaCl_2_. Although the presence of NaCl in solutions did not result in activation of the cellular nucleases, the increase of NaCl concentrations resulted in the decrease of the rate of the signal growth during the real-time measurements. The growth of the signal was completely stopped in solutions containing 150 mM NaCl.

As further experiments showed that the solution containing a combination of 2 mM CaCl_2_, 50 mM NaCl, 10 mM Tris–HCl, pH7.4 and 1 mM EDTA (NaCa buffer) does not activate or only marginally activates the nucleases in cell lysates but sufficiently stabilizes the used ON probes (figure 2*d*), this buffer was used in the subsequent experiments. Although we observed only a low effect of nucleases in our experiments, other systems can embody distinctly higher nuclease activity. To decrease the impact of nucleases, the signal from sensors with thymine was subtracted from the signal of m-sensors with probes containing uracil before the calculation of the initial rate of the signal growth.

#### 3.1.2. SPR sensor

The SPR sensor used an identical double-stranded arrangement, where an ON duplex was attached to the streptavidin-coated sensor surface via biotin. For SPR sensor principle and assay optimization, see Material and Methods, section 2.3 and electronic supplementary data, methods 1.4.

**Figure 3.**
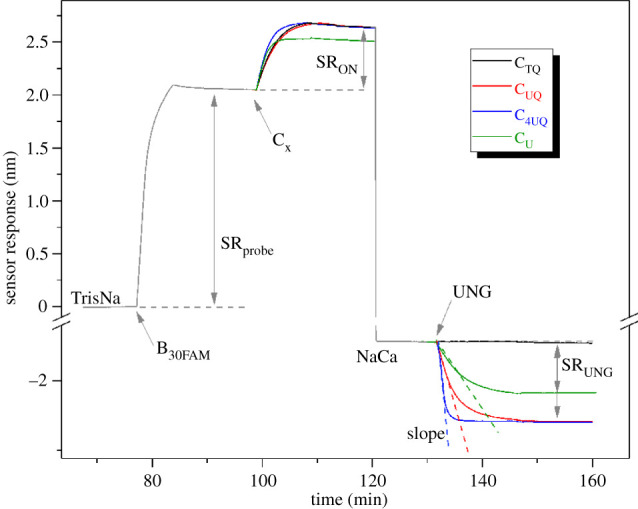
The temporal SPR sensor response. The temporal sensor response to the binding of biotinylated anchoring ON, B_30FAM_, to a streptavidin-coated SPR chip, and to the hybridization and subsequent dissociation of four different complementary ONs: C_TQ_ (black), C_UQ_ (red), C_4UQ_ (blue) and C_U_ (green).

As a result of the UNG cleavage activity, the ON duplex was destabilized and the complementary ON was released, causing a decrease in the sensor response. The cleavage (and corresponding ON release) was found to stop when the UNG solution was replaced by NaCa buffer. A typical sensorgram showing all the main steps of the SPR detection assay is presented in figure 3.

The UNG activity was characterized in terms of cleavage efficiency (CE) and ON release rate (RR). CE is defined as the ratio of dissociated to bound complementary ONs, and was calculated as CE = SR_UNG_/SR_ON_, where SR_UNG_ and SR_ON_ denote the absolute value of the sensor response to the dissociation and binding of the complementary ON, respectively. RR is defined as the maximum negative slope of the dissociation curve of a complementary ON. All complementary ONs used with the SPR sensor were of similar molecular weight (difference within 6%), hence the characterization of the effect of different probe designs on the UNG activity is made possible via respective CE and RR values.

The SPR assay was optimized in terms of the surface density of the anchoring ONs (SDe) and ON duplexes (DD) to provide a high UNG cleavage efficiency and a sufficiently high sensor response. In the following experiments, an SDe of 2.0 × 10^12^ anchoring ON cm^−2^ and DD of 1.3 × 10^12^ duplexes cm^−2^ were used in order to provide reproducible experimental conditions, respectively. More details on the SPR assay optimization are provided in electronic supplementary material, methods 1.4 and figure S4.

It should be noted that under given experimental conditions all ON probe designs formed stable duplexes. Experiments with all oligonucleotide probe designs were repeated at least three times and the reproducibility of respective CE and RR values was higher than 90% and 80%, respectively. The specificity of the SPR assay was verified by using the control complementary ON (C_TQ_). As shown in figure 3 (black curve), no dissociation occurred upon the injection of UNG to the sensor surface coated with B_30FAM_C_TQ_ probe.

### 3.2. Structural aspects of the probes' construction

#### 3.2.1. Position of 6-FAM and BHQ-1 in probes significantly affects the activity of bacterial UNG, DNase I and exonuclease III

**Figure 4.**
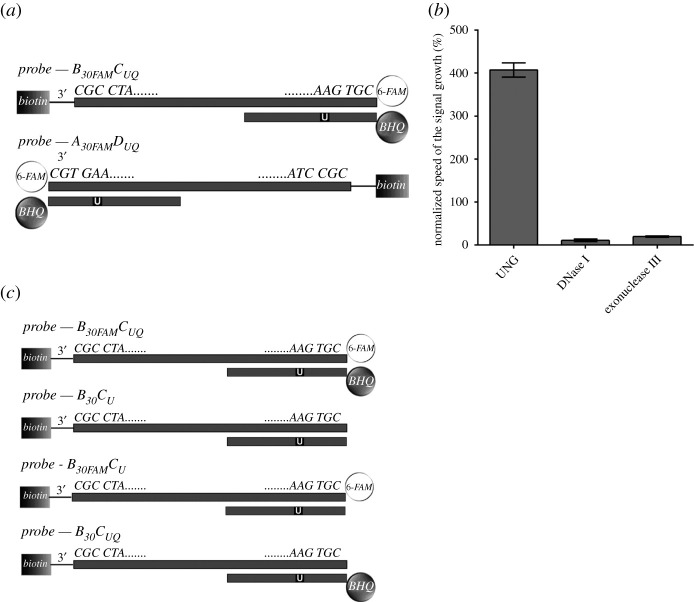
The impact of the 6-FAM fluorochrome and BHQ-1 quencher position in the ON duplex. (*a*) The scheme of the used probes B_30FAM_C_UQ_ and A_30FAM_D_UQ_. (*b*) The results of analysis of the impact of the position of 6-FAM and BHQ-1. The speed of the signal growth for m-sensors containing B_30FAM_C_UQ_ probe was normalized to the speed of the signal growth calculated for the A_30FAM_D_UQ_ probe (equal to 100%) and the given enzyme and plotted. The data are shown as the mean ± s.d. (*c*) The scheme of the used ON probes for the determination of the impact of 6-FAM and BHQ-1 on the glycosylase activity by an SPR chip.

For the analysis of the impact of the position of 6-FAM and BHQ-1 on the activity of bacterial UNG, DNase I and exonuclease III, ON probe arrangement with mutually inverted sequences and with 6-FAM and BHQ-1 on mutually opposite ends was used (figure 4*a*). The applied concentration of bacterial UNG was 40 mU ml^−1^, DNase I 0.5 U ml^−1^ and exonuclease III 25 U ml^−1^.

The growth of the signal was *ca* 4 times faster in solutions with m-sensors containing B_30FAM_C_UQ_ probe (6-FAM on 5′ end) than with m-sensors containing A_30FAM_D_UQ_ probe (6-FAM on 3′ end) if bacterial UNG was used (figure 4*b*). On the contrary, the growth of the signal in solution containing m-sensors with B_30FAM_C_UQ_ probes was *ca* 9 times (DNase I) and 5 times (exonuclease III) slower than in the solutions containing m-sensors with A_30FAM_D_UQ_ probes (figure 4*b*).

Obtained results showed that the ON probe arrangement with the fluorescent label at the 5′ end (B_30FAM_C_UQ_) allows for higher glycosylase activity and concurrently for lower nuclease activity than the system with the fluorescent label at the 3′ end (A_30FAM_D_UQ_). In this respect, it seems that the position of 6-FAM and BHQ-1 in ON probes affects both the uracil DNA glycosylase and the nuclease activity. The very low sensitivity of the system based on B_30FAM_C_UQ_ probes to nuclease activity is profoundly important for the application of m-sensors for the measurement of uracil DNA glycosylase activity in complex mixtures such as cell lysates. Therefore, we used in the subsequent experiments B_30FAM_C_UQ_ probes for measurement of uracil DNA glycosylase activity if not stated otherwise.

#### 3.2.2. The presence of 6-FAM and BHQ-1 in probes does not decrease the activity of bacterial UNG

SPR sensor was used to analyse the impact of 6-FAM and BHQ-1 on the activity of bacterial UNG. An overview of the probes used is in figure 4*c*. The calculated CE and RR values (table 3) indicated that the presence of 6-FAM and BHQ-1 does not inhibit the glycosylase activity. Instead, the presence of dyes supports the UNG activity, probably due to the steric effect that is conducive to ON duplex dissociation. In particular, the dissociation was more promoted by BHQ-1 than by 6-FAM, which correlates well with their respective molecular weights of 677 Da (BHQ-1) and 376 Da (6-FAM).

**Table 3.** Calculated cleavage efficiency (CE) and ON release rate (RR) for probes with and without 6-FAM fluorochrome and/or BHQ-1 quencher.

probe	CE (%)	RR (10^−3^ nm. min^−1^)
B_30FAM_C_UQ_	93 ± 4	−154 ± 19
B_30_C_U_	65 ± 8	−41 ± 9
B_30FAM_C_U_	85 ± 4	−94 ± 21
B_30_C_UQ_	83 ± 5	−146 ± 25

#### 3.2.3. The quadruple number of uracils leads to around a twofold increase in the sensors’ response

We compared the glycosylase activity with respect to the probes containing either four or one uracil (figure 5*a*). The presence of four uracils resulted in a more than twofold increase of the speed of signal growth of m-sensors and this ratio was independent of the UNG concentration (figure 5*b*). A similar trend was observed in the case of the SPR sensor, where the ratio of the RR between a probe with four and one uracil was 2.52 (figure 3; table 4, first two rows). It pointed to the importance of the position of uracil with respect to the effectivity of its cleavage by UNG.

**Table 4.** Calculated cleavage efficiency (CE) and ON release rate (RR) for different probe designs.

probe	CE (%)	RR (10^−3^ nm. min^−1^)
B_30FAM_C_UQ_	93 ± 4	−154 ± 19
B_30FAM_C_4UQ_	102 ± 8	−388 ± 13
B_14FAM_C_UQ_	98 ± 4	−143 ± 5
B_30FAM_C_UQ_F_break_	39 ± 4	−18 ± 3
B_30U_	98 ± 5	−10 ± 3

The sensitivity of m-sensors with probes with four uracils was higher as well. We compared the coefficients of determination (*R*^2^) for the time course of the signal in serially diluted solutions of bacterial UNG. We observed the significant decrease of *R*^2^ value under 0.9 in the case of m-sensors containing probes with four uracils if the concentration of UNG was 3.125 mU ml^−1^ and in the case of m-sensors containing the probes with one uracil if the concentration of UNG was 6.25 mU ml^−1^ (electronic supplementary material, figure S6). Therefore, the detection limit was estimated to be around 12 mU ml^−1^ for m-sensors with one uracil and 6 mU ml^−1^ for m-sensors with four uracils.

Detection limits and features of chosen fluorescence and colorimetric methods are shown table 5.

**Table 5.** Comparison of chosen methods used for glycosylase activity measurement.

method type	principle	detection limit	compatibility with cell lysates	real-time	typical protocol length	measurement
fluorescence (the developed assay; this work)	Fluorescence measurement is accompanied by the dissociation of linear ON probes conjugated with fluorochrome and fluorescence quencher. Probes are anchored on magnetic particles.	6 mU ml^−1^ in well-defined solutions 1–2 µg of overall protein in nuclear lysates.	yes	yes	one-step protocol, 20–40 min (including measurement)	plate reader, spectrophotometer or fluorescence microscope
surface plasmon resonance (the developed assay; this work)	Measurement is accompanied by the dissociation of the non-labelled linear ON probes. Probes are anchored on SPR chip.	n.a.	no	yes	one step protocol, 20–30 min	SPR-based sensor
fluorescence [30]	Measurement of fluorescence is accompanied by the dissociation of fluorochrome-labelled hairpin ON probe after nuclease treatment and graphene oxide induced quenching of fluorescence of non-cleaved probe.	5 mU ml^−1^	n.a.	n.a.	three-step protocol, approx. 70 min (measurement not included)	plate reader or spectrophotometer
fluorescence [28]	Measurement of fluorescence is accompanied by the dissociation of molecular beacons.	n.a.	yes	yes	one-step protocol, 20–40 min (including measurement	plate reader or spectrophotometer
fluorescence [34]	Sngle-molecule detection after magnetic separation of fluorescently non-labelled probes and endonuclease IV-assisted signal amplification.	0.01736 mU ml^−1^	n.a.	n.a.	three-step protocol, approx. 120 min (measurement not included)	fluorescence microscopes convenient for single-molecule detection (e.g. TIRF)
luminescence [32]	Detection of luminescence after formation of G-quadruplex from non-labelled double-stranded probe and binding of small organic molecule (DID-VP) to ON.	5 mU ml^−1^	n.a.	n.a.	two-step protocol, >30 min (measurement not included)	fluorescence plate reader or spectrophotometer
colorimetric [33]	Detection of colorimetric signal after formation of G-quadruplex from non-labelled double-stranded probe and signal generation by peroxidase activity of quadruplex and hemin.	8 mU ml^−1^	n.a.	n.a.	three-step protocol, approx. 75 min (measurement not included)	plate reader, smartphone or spectrophotometer
colorimetric [31]	Detection of colorimetric signal produced by peroxidase activity of quadruplex and hemin after dissociation of non-labelled double-stranded probe and Toehold-mediated strand displacement circuit.	6 mU ml^−1^	n.a.	n.a.	four-step protocol, approx. 75 min (measurement not included)	plate reader or spectrophotometer

#### 3.2.4. The effect of the length of the anchoring oligonucleotide

To address the impact of the length of the ON probes on uracil DNA glycosylase activity, biotinylated anchoring ON with 30 or 14 nt were used (figure 5*c*). In the case of bacterial UNG and m-sensors, the shortening of the length of the anchoring oligonucleotide from 30 nt to 14 nt resulted in around a 40% decrease of m-sensors response (figure 5*d*). In the case of nuclear lysates, the different length of the biotinylated anchoring ON entailed no significant change (less than 2.5%) in the glycosylase activity (figure 5*d*). When the effect of the probe length was investigated using SPR sensor, almost no difference in the UNG activity was observed. Both CE and RR values did not differ by more than 7% (table 4) for probes with long (B_30FAM_C_UQ_) and short (B_14FAM_C_UQ_) biotinylated anchoring ONs.

Although the reason for these differences is not clear, it points to the high number of factors exhibiting an effect on uracil DNA glycosylase activity and indicates that the length of the probe can also have an important impact on its activity.

#### 3.2.5. DNA breaks induce significant decrease of uracil DNA glycosylase activity

**Figure 6.**
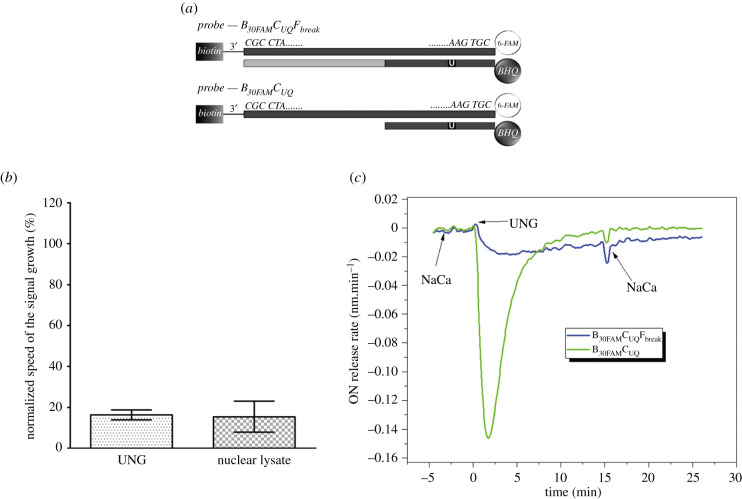
Impact of the break in the strand containing uracil on glycosylase activity. (*a*) Scheme of probes for the measurement of the impact of the break in the strand containing uracil on the glycosylase activity. (*b*) Results of the measurement of the impact of the break in the strand containing uracil on the glycosylase activity. M-sensors were incubated during the measurement with either UNG (40 mU ml^−1^) or nuclear lysate of HeLa cells (2 µg ml^−1^ of the overall protein). The speed of the signal growth was normalized to the speed of the signal growth in control samples with B_30FAM_C_UQ_ probe equal to 100%. The data are shown as the mean ± s.d. (*c*) First derivatives of curves corresponding to the sensor response to the dissociation of complementary oligonucleotides upon the injection of UNG; the sensor surface was functionalized with two different probes: B_30FAM_C_UQFbreak_ (violet), B_30FAM_C_UQ_ (green).

The effect of the introduction of the break into the DNA strand near the uracil was studied by the m-sensors with B_30FAM_C_UQ_ probe with additional short F_break_ ON tightly adjacent to the chain containing uracil (figure 6*a*). This arrangement led to the strong inhibition of glycosylase activity in both buffer with bacterial UNG and nuclear lysates (figure 6*b*). The speed of the signal growth reached only approximately 16% of the speed of the control probes without F_break_ ON in both systems. The same effect was observed when SPR method was used (figure 6*c*), where the CE and RR value decreased to 39% and 18%, respectively (table 4).

Lower glycosylase activity observed for m-sensors in buffer with m-sensors could be connected to the missing single-strand area in the ON probe. This would be in agreement with the previous results with short B_14FAM_ anchoring ON which also provided lower glycosylase activity (cf. figure 5*d*). However, such effect was observed neither with the SPR sensor in buffer nor with the m-sensor in nuclear lysates (cf. figure 5*d* and table 4). It strongly indicates that the break in DNA, but not the omission of single-stranded DNA, plays an important role in reducing of UNG glycosylase activity. Although any functional interpretation of this phenomenon is highly speculative, it can serve for preventing a collision with DNA replication accompanied by DNA breaks formation or a collision with a repair system of these breaks.

### 3.3. Analysis of single-stranded arrangement by means of SPR sensor

A direct study of the interaction between UNG and single-stranded ON probe (B_30U_) was performed using an SPR sensor. In this particular case, the CE was calculated as CE = SR_UNG_ * M_probe_/SR_probe_ * M_U_, where SR_UNG_ and SR_probe_ denote the absolute value of the sensor response to the dissociation of the complementary ON and to the binding of anchoring ON, respectively. M_probe_ and M_U_ are the molecular weights of the biotinylated anchoring ON and uracil, respectively. Due to the low molecular weight of uracil (approx. 40 times lower than the molecular weight of complementary ON), its release due to the UNG cleavage generated only a small sensor response. In spite of that, the value of CE (table 4) can be directly compared with those obtained from the experiments exploiting the release of complementary ON. However, a direct comparison of RR is not straightforward and thus the RR value only serves as a qualitative indicator of the uracil release.

### 3.4. M-sensors can be used for the analysis of single-stranded substrates if the competitive arrangement is used

As the construction of m-sensors makes the direct analysis of single-stranded DNA containing uracil target base not possible, we used m-sensors in the competitive arrangement instead. We supposed that the presence of UNG substrate will decrease the signal of m-sensors and such decrease will be proportional to the affinity of UNG to the substrate.

**Figure 7.**
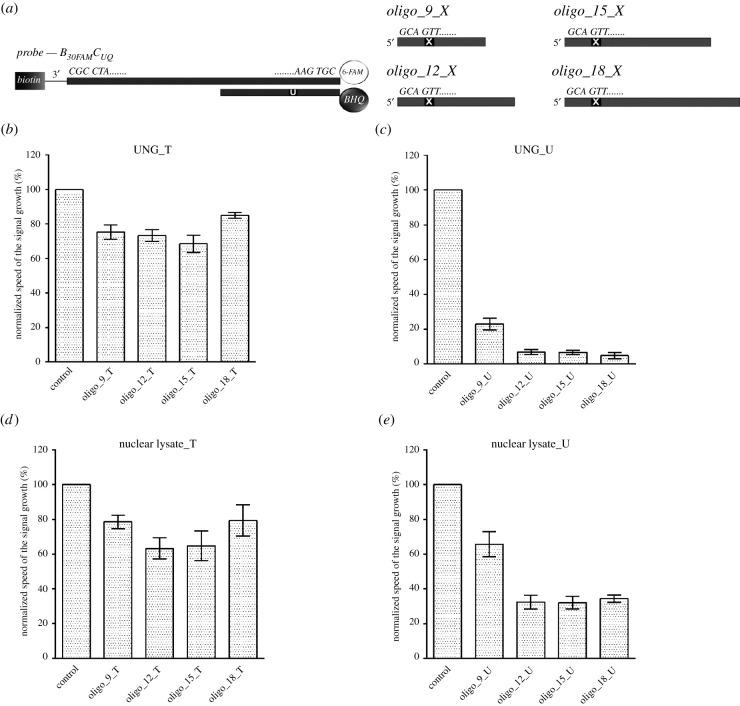
The impact of the addition of single-stranded ONs with various length. (*a*) Scheme of the used probe (B_30FAM_C_UQ_) anchored to m-sensors and the single-stranded ONs used for the analysis of their impact on m-sensor response. X = uracil or thymine. (*b*–*e*) Results of the measurement of the impact of the addition of the single-stranded ONs with various length with thymine (T) or uracil (U) on the speed of the signal growth. M-sensors with B_30FAM_C_UQ_ probe were during signal measurement incubated with 1 µM solution of ONs with thymine or with uracil and either with UNG (40 mU ml^−1^) or nuclear lysate of HeLa cells (2 µg ml^−1^ of the overall protein). In the case of control samples, the solution of ON was not added. The speed of the signal growth was normalized to the speed of the signal growth in control samples. The data are shown as the mean ± s.d.

We analysed the impact of the addition of competitive ONs of different lengths (table 2 and figure 7*a*) with uracil or thymine to the reaction mixture containing m-sensors with B_30FAM_C_UQ_ probe (figure 7*b*–*e*). The analysis was performed both with bacterial UNG and nuclear lysates. Although the addition of ONs with thymine led to a decrease of the speed of the fluorescence signal growth in the case of bacterial UNG, the impact of the addition of ONs with uracil was much stronger (figure 7*b*,*c*). Simultaneously, the strong relation between the ON length and m-sensors reaction was obvious. The addition of 9-mer ON with uracil led to a roughly 4.4-fold decrease of the speed of the fluorescence growth, while the addition of 18-mer ON to an almost 21-fold decrease compared to samples without any competitive ON addition. In this respect, the addition of ONs with thymine resulted to only around 1.2–1.5 times lowering of the uracil DNA glycosylase activity.

When the nuclear lysates were used, we also observed a higher decrease of the speed of fluorescence signal growth in samples with ONs with uracil than with thymine (figure 7*d*,*e*). However, the difference was significantly lower. The addition of 9-mer ON led to a roughly 1.5 times decrease of the speed of the signal growth, whereas the addition of 12-mer ON to a roughly 3 times lower speed. Further increase of the ON length did not lead to significant additional change of the speed of the signal growth. Concerning the ONs with thymine, the decrease was *ca* 1.3–1.6-fold.

The difference between bacterial UNG and nuclear lysates can result from the high heterogeneity of lysates and the presence of components binding to the ONs preventing their ability to be effectively processed by uracil glycosylases. Such interpretation is in accordance with our finding that the free probes are an ineffective tool for the detection of uracil DNA glycosylase activity in cellular lysates (see the next chapter).

### 3.5. M-sensors versus free probes

The anchored ON probes should more closely reflect the real conditions given by the relatively immobilized nuclear chromatin than the free probes. However, it was crucial to investigate the effect of ON probe immobilization on the efficiency of the assay. Therefore, we compared the glycosylase activity measured by m-sensors, free probes and molecular beacons prepared according to Maksimenko *et al.* [28] (for the scheme see electronic supplementary material, figure S7) for both bacterial UNG and glycosylases naturally present in nuclear lysates using the same conditions. In these experiments, 0.5 µg of m-sensors (approx. 0.6 pM solution of the probe) or 0.6 pM of the free probes or molecular beacons per well of the 384-well plate were used.

#### 3.5.1. Free probes provide less reliable data than m-sensors in the case of bacterial UNG

Experiments with m-sensors containing B_30FAM_C_UQ_ and B_30FAM_C_TQ_ probes, free B_30FAM_C_UQ_ and B_30FAM_C_TQ_ probes and corresponding molecular beacons either with uracil or thymine showed that the time course of the signal was very similar for uracil-containing probes (figure 8*a*). However, the significant decrease of the signal was observed at control free probes and molecular beacons with thymine. No such decrease was observed if m-sensors were used.

The reason of the differences between m-probes and free probes and molecular beacons can probably be attributed to the association of the used free probes and molecular beacons to the well surface and consequently to the decrease the effectivity of uracil cleavage and/or decrease of the probe concentration in the measured zones.

#### 3.5.2. M-sensors are a more suitable tool for the measurement of uracil DNA glycosylase activity in cell lysates than free probes and molecular beacons

We tested m-sensors, free probes and molecular beacons for the measurement of uracil DNA glycosylase activity naturally present in nuclear lysates. In this case, the differences between the used systems were fundamental (figure 8*b*). In the case of m-sensors, the speed of the signal growth was around 30 times higher for probes with uracil than for probes with thymine. On the contrary, zero or even negative signal growth rate was achieved with molecular beacons or free probes. Control molecular beacons and free probes with thymine provided decreasing temporal fluorescence signal (figure 8*b*).

The reason for such significant differences between immobilized probes and free probes and molecular beacons is not clear. One possibility could be the high ability of free probes and molecular beacons to bind the cellular residues present in lysates causing the inability of glycosylases to cleave off the target uracil from the ON probes and/or to bind to the well surface *per se* and via these residues and consequently the decrease of the probe concentration in the measured zones. This interpretation is in agreement with the results obtained in experiments with bacterial UNG. Independently of the reason of this finding, m-sensors with immobilized ON probes could be considered as a more suitable approach for the determination of glycosylase activity in nuclear lysate than approaches based on free probes and molecular beacons. As we did not observe similar decrease of the signal in the case of m-sensors, we did not further deal with the reason of the decrease of the signal in the case of the free probes and molecular beacons.

When we performed similar experiments with SPR sensor assay, it was not possible to observe the specific response due to the high non-specific interaction of components of the lysate with the sensor surface. Therefore, the SPR sensor was not employed in the cell lysate study. On the other hand, this result indicates that the binding of various components to the well surface can be an important factor effecting the result of the assay.

### 3.6. Activity of cellular glycosylases is significantly lower in cytoplasmic lysates than in nuclear lysates and depends on the method of lysate preparation

We compared the glycosylase activity in cytoplasmic and nuclear lysates prepared by two different methods. The first method was based on the use of the Dounce homogenizer, the second one on the use of the detergent Triton X-100. Regardless the method of lysate preparation, lower glycosylase activity was observed in cytoplasmic lysates than in nuclear lysates. The homogenization by the Dounce homogenizer provided slightly higher glycosylase activity in nuclear lysates and significantly lower activity (around 50%) in cytoplasmic lysates of HeLa cells comparing to Triton X-100 treated lysates (electronic supplementary material, figure S8a).

Post-incubation of lysates with Triton X-100 increased the glycosylase activity in cytoplasmic lysates but not in nuclear lysates (electronic supplementary material, figure S8b). The increased glycosylase activity in the Triton X-100 treated cytoplasmic lysates can be a consequence of the destruction of mitochondria and release of their content (mitochondrial glycosylase—UNG1) into cytoplasmic lysates by Triton X-100. It corresponds well to the amount of UNG found in the respective lysates as estimated by western blot. In this case, samples are treated by SDS during their preparation for western blot. This step can result in the mitochondria destruction and release of UNG1. In this respect, a similar ratio between the amount of UNG and nuclear and cytoplasmic lysates independently of the method of preparation was observed (electronic supplementary material, figure S8c).

These results indicated that the approach based on Triton X-100 is the preferential choice for the measurement of UNG1 (mitochondrial) activity while the method based on Dounce homogenization is preferential for the measurement of UNG2 (nuclear) activity.

### 3.7. The decrease of UNG content in HeLa cells significantly lowers the m-sensor response

In order to verify the specificity of the method in cell lysates, m-sensors were used for the analysis of glycosylase activity in nuclear lysates of HeLa cells with an intentionally decreased level of UNG after siRNA treatment (figure 8*c*). The control cells were treated with the control siRNA which does not inhibit the expression of UNG. The decrease of the amount of UNG in the nuclear lysates was simultaneously monitored by western blot (figure 8*d*). The speed of the signal growth in cells treated with UNG-siRNA was approximately 3.7 times lower than in the control cells. The integral density of the signal from western blot was about 2.3 times lower than in the cells treated with control siRNA. It pointed to either high specificity of m-sensors for the glycosylase activity mediated by UNG or very low activity mediated by other uracil DNA glycosylases. Such a correlation was also in accordance with the mutual ratio of UNG amount determined by western blot and the results of glycosylase activity in nuclear and cytoplasmic lysates prepared by homogenization. The ratio between nuclear and cytoplasmic lysates was about 2 for both cases (see electronic supplementary material, figure S8a–c).

## 4. Conclusion

In the study presented, we described two systems for the real-time measurement of glycosylase activity. They are based on the linear oligonucleotide probes anchored on magnetic particles (m-sensors) or on SPR chips and are performed in one step. The assay length was typically 20–40 min. The system based on SPR chip is advantageous for the determination of uracil DNA glycosylase activity in a well-defined solution as no preparation of fluorescently labelled probes is required. On the other hand, the presence of fluorochrome or fluorescence quencher did not result in the decrease of the signal growth. Since the flow system is used, it allows very fast change of the individual solutions and thus, it is suitable for kinetic studies.

The system based on m-sensors was successfully used for the real-time uracil DNA glycosylase activity determination both in the well-defined solutions of the purified enzyme and in cell lysates. In this respect, plate readers or fluorescence microscopes can be used for signal measurement. We showed that m-sensors can be used for the determination of a nuclease activity as well.

The sensitivity limit for bacterial uracil DNA glycosylase and m-sensors with the probes with one uracil was 12 mU ml^−1^. The sensitivity limit for m-sensors with the probes with four uracils was *ca* 6 mU ml^−1^. In the case of cellular lysates, the sufficient concentration of overall protein was around 1–2 µg ml^−1^. The comparative study showed that in contrast to the free probes or molecular beacons, anchoring the probes provides significantly more reliable results if glycosylase activity is determined in cell lysates.

The used system allows also the analysis of the role of structural aspects of uracil repair. In this respect, we showed that the break of the phosphodiester linkage in the chain carrying the uracil leads to a strong decrease of the ability of uracil DNA glycosylases to remove uracil from the probe. In addition, although the measurement is based on the double-stranded probes, if the competitive arrangement is used, the developed system provides possibility to study the aspect related to the cleavage of uracil in the single-stranded probe.

## Data accessibility

All data are included in this article and its electronic supplementary material [45].
